# Transfusion as an Inflammation Hit: Knowns and Unknowns

**DOI:** 10.3389/fimmu.2016.00534

**Published:** 2016-11-29

**Authors:** Olivier Garraud, S. Tariket, C. Sut, A. Haddad, C. Aloui, T. Chakroun, S. Laradi, F. Cognasse

**Affiliations:** ^1^Faculty of Medicine of Saint-Etienne, University of Lyon, Saint-Etienne, France; ^2^Institut National de la Transfusion Sanguine, Paris, France; ^3^Hôpital du Sacré-Coeur, Beirut, Lebanon; ^4^Centre de Transfusion Sanguine, Sousse, Tunisia; ^5^Faculty of Pharmacy, University of Monastir, Monastir, Tunisia; ^6^Etablissement Français du Sang Rhône-Alpes-Auvergne, Saint-Etienne, France

**Keywords:** inflammation, transfusion, allergy, blood components, leukocytes, platelets, erythrocytes, alloimmunization

## Abstract

Transfusion of blood cell components is frequent in the therapeutic arsenal; it is globally safe or even very safe. At present, residual clinical manifestations are principally inflammatory in nature. If some rare clinical hazards manifest as acute inflammation symptoms of various origin, most of them linked with conflicting and undesirable biological material accompanying the therapeutic component (infectious pathogen, pathogenic antibody, unwanted antigen, or allergen), the general feature is subtler and less visible, and essentially consists of alloimmunization or febrile non-hemolytic transfusion reaction. The present essay aims to present updates in hematology and immunology that help understand how, when, and why subclinical inflammation underlies alloimmunization and circumstances characteristic of red blood cells and – even more frequently – platelets that contribute inflammatory mediators. Modern transfusion medicine makes sustained efforts to limit such inflammatory hazards; efforts can be successful only if one has a clear view of each element’s role.

## Introduction

Historically, inflammation was viewed as the compendium of all four stigmas: “*rubor, calor, dolor*, and *tumor*”; this concept fits well with the theory of humors; bloodletting – and surrogates (e.g., leeches and suction cups) – have long been applied to treat, if not cure, inflammation symptoms. As a matter of fact, iron depletion caused by bloodletting happened to alter bacterial growth and ameliorate certain disease conditions, as already observed by Tissot in 1761 (1). The Hippocratic theory of humors was probably the first to introduce the relationship between blood and inflammation, though using wrong descriptors. In its earliest days, transfusion was clearly associated with acute inflammation, though the connection was not acknowledged as such: indeed, the very first reported serious adverse events (SAEs) of “modern” transfusion in the early twentieth century were dual in nature: first, immune-hematological [i.e., antigen–antibody (ABO)] conflicts, and second, blood-borne and blood-transmitted infections, such as syphilis and malaria (2). Both conditions – presenting as very severe – were later on acknowledged as being dominated by cytokine storms and standing for acute inflammatory reactions (often lethal) (3, 4).

The concept of inflammation has been largely revisited by modern internal medicine; series of autoimmune and auto-inflammatory diseases have thus been acknowledged. No organ-specific disorder is actually beyond the scope of the large clinical inflammation spectrum, since a number of neurological disorders (5) – as well as many cardiovascular lesions especially the atheroma plaque deposit (6) – are inflammation stigmas. The causality of inflammation in organ-specific lesions is being questioned, but combinations of genetic predisposition, lifelong hygienic habits, other environmental factors, and infectious triggers are commonly evoked. For decades now, clinical inflammation has not been restricted to acute Hippocratic symptoms and is acknowledged to present as more subtle symptoms of varying degrees.

We believe two major achievements have helped reconsider clinical inflammation as it may apply to transfusion medicine and cell, tissue, and organ transplantation. Neither was intended to apply to this discipline; however, the first is the (re)discovery of the danger signal theory, as proposed by P. Matzinger at the NIAID, NIH, in the 1990s, after its seminal conceptualization by E. Metchnikoff at the Pasteur Institute 100 years earlier. This discovery is basic to immunology and helps reframe the reading of immunology (7). The second is the conceptualization of the microbiota’s role in immunity – initially presented as governing what Ph Sansonetti (at the Pasteur Institute in Paris) called “war and peace” at the mucosal surfaces. This concept helped show that inflammation spans the whole spectrum, from physiology to pathology (8–10). It has since been suggested that healing (e.g., of tissue attrition or organ lesions) is the ultimate step of inflammation (11, 12).

## Transfusion and Inflammation: From Bedside to Bench

From the bedside, one can consider two periods in relation to transfusion-related hazards, especially inflammation. The initial period concerns acute symptoms of SAEs: inflammation is observed among other symptoms such as shock. Those accidents were principally reported with reference to their major cause(s): the ABO conflict, transfusion-transmitted bacterial, viral, or parasitic infections, and allergy. In 1983, a novel cause of transfusion-transmitted SAE was described: *transfusion-related acute lung injury* (TRALI) (13–15). Interestingly, this SAE is ascribed to a dual cause: an Ag/Ab conflict – within the human leukocyte antigen (HLA) or, more rarely but more severely, the human neutrophil antigen (HNA) systems – and an inflammatory layer: sepsis, stress, etc. Besides conflicting Abs (when identified, i.e., in two out of every three cases on average), the principal actors are leukocytes recruited or residing in lung capillaries. The TRALI concept prompted a reinvestigation of SAEs in transfusion and acknowledgment of serious inflammatory cases. This is also true for allergy: though one cannot exclude the possibility of pathogenic IgE transfer, it is rather felt that such an occurrence cannot account for one-third of adverse events (AEs) varying in severity (16, 17). Transfusion allergy is, in general, considered to present like allergy, though it is not believed to have a link with atopy or involve allergens or Abs specific to allergens. It is recognized as one of the most frequent inflammatory consequences of transfusion (18).

In summary, despite this is over-simplistic, one may acknowledge that inflammation symptoms manifested by a transfused patient and in relation with the transfusion process has two principal causes: it is either due to the transfer of pathogenic material collected from the donor or it is due to a conflict between high affinity receptors found on the recipients’ cells or plasma molecules and ligands brought by the transfused component.

The majority of AEs in patients receiving blood (recipients) manifest either allergy or febrile non-hemolytic transfusion reactions (FNHTRs), both being clearly inflammatory conditions (19). Leukocytes transferred with blood were ascribed to as the principal causes of TT inflammation. Systematic “leucoreduction” – often inappropriately, but nevertheless officially, termed “leucodepletion” – was proposed at the start of the millenium by many countries or blood transfusion systems. However, leucoreduction has neither been become recommended nor a mandatory practice for mitigating inflammatory responses but is instead used to limit transfusion-transmitted viral risks as many “serious transfusion-associated viruses” are intracellular. Leucoreduction was principally aimed to reduce the risk of transmitting the Creutzfeldt–Jakob prion (20). Veterans of transfusion medicine very well recall the time when every single transfused patient was “shaking and heating,” manifesting common symptoms that were subsequent to the therapy and introduced as such to patients (when patients happened to receive information). Pre-storage leucoreduction was then acknowledged to have largely improved comfort and safety in patients, suggesting a deleterious role for leukocytes (21, 22). When leucoreduction is performed post-storage (e.g., at the bedside, prior to the infusion of the blood component), inflammatory manifestation is intermediate, largely suggesting that not only leukocytes but also their secreted content play a role in the transfusion inflammation pathophysiology (23).

However, as the transfused patient profile changed, more and more recipients benefited from platelet components (PCs). This major change took place more or less at the same time as the implementation of systematic hemovigilance, and it soon became obvious that PCs – though representing no more than 10% of issued blood components – provide between one-quarter to one-half of reported AEs (24). This means that leukocytes were not the only cells associated with transfusion-associated inflammation.

Another population of patients benefiting from frequent transfusion episodes, sickle-cell disease patients, led to an important discovery: first, they manifest complex hemolytic reactions that involve activated complement and present as essentially inflammatory (25) and second, they are subjected to the most frequent rate of alloimmunization among tracked cohorts of transfused patients (26). This prompted specialists to also examine the inflammatory potential of stored erythrocytes.

## The Major Identified Causes of Transfusion-Associated Inflammation

There is good evidence in favor of an undesirable role for residual leukocytes in transfused patients; blood banks can get rid of such residual leukocytes with a high degree of efficacy by using filtration methods. Leucoreduction is highly recommended by the European Community and the American Association of Blood Banks with a target of no more than 10^6^ residual leukocytes per blood component after filtration (27, 28); most pre-storage methods allow scores of leucoreduction ranging between 2 and 5 × 10^5^ residual leukocytes per component (29). However, clinical observations suggest that other constituents of pre-stored and leucoreduced cellular blood components still lead to some inflammatory manifestations in patients.

To simplify and summarize, pro-inflammatory factors in labile blood components fall into one of the following four categories: (1) infectious pathogens transmitted by blood that cause bacterial sepsis, acute or chronic viral infection, or acute parasitic infection (and often hemolysis); (2) pathogenic, undesirable, Abs (causing hemolysis when encountering target Ags, especially when capable of binding complement; causing TRALI, depending on circumstances or predisposition; causing Reagin-mediated allergy; and causing a number of non-hemolytic situations, now ascribed to FNHTRs); (3) leukocytes and their content, and especially their high loads of pro-inflammatory cytokines, chemokines, and the like, collectively termed *biological response modifiers* (BRMs); and (4) (pro)inflammatory material linked to platelet and erythrocyte pathophysiology, especially when cellular blood components [PCs and packed red blood cell components (pRBCCs)] are stored over time and undergo so-called storage lesions, which consist of extracellular vesicle emission and the freeing of membrane-bound molecules and intracellular content, either iron (erythrocytes) or BRMs (platelets). It is not unusual that extracellular vesicles are called microparticles.

The first two categories are chiefly inflammatory, involving two principal mechanisms: first, the triggering of a cytokine storm, with broad consequences for all systems, exposing the patient to multivisceral failure and severe central neurologic disorders. Second, if erythrocytes are ultimate targets of the Ab or infectious pathogens, there is acute hemolysis with obvious consequences. This essay will not further discuss such cases. Neither will it discuss the specific case of bacterial contamination of PCs, largely related to the storage temperature of 22 ± 2°C; 18.5 severe cases per million PCs delivered are recorded annually according to the latest French hemovigilance records; and one such case happens to be lethal, on average (30).

The latter two categories reveal that inflammation is not only the result of substantial levels of BRMs secreted by leukocytes, platelets, or lysed erythrocytes but also of products secreted as a consequence of cell–cell encounters after the blood component has been transfused. Cell–cell interactions occur mainly between (i) donor transfused cells and recipient circulating cells and (ii) donor transfused cells and recipient vascular endothelium cells. Transfusion is a dynamic process, but it is often regarded as the passive infusion of therapeutic components (31). This view is misleading and a source of errors for the interpretation of transfusion-associated inflammation.

## Models of Transfusion-Associated Inflammation Hit

This section will address three main issues: (1) how transfusion can act as a stress for the recipient, subsequently triggering an immune defense; (2) how blood components can present with varying degrees of stress signals, accompanied by pathogenic storage lesions; and (3) how the stage is set for a recipient’s adaptive immune response to donor cell Ags. These three points parallel three major aspects of the transfusion process or chain: donor-linked characteristics, additional pathogenic steps during blood component production, and recipient-linked characteristics.

### Transfusion as a Stress and Donor-Linked Characteristics Account for Recipients’ Inflammatory Symptoms

Transfusion is an unnatural process in the sense that the exchange of body parts between individuals – other than mothers and their embryos or fetuses – is not part of the human evolutionary program. Each individual’s blood has potentially unique biological characteristics. Thus, when foreign cellular material, is tentatively grafted into a recipient, the latter identifies it as foreign and potentially dangerous, even when it has a therapeutic purpose. Indeed, many studies have shown that platelets express a large variety of pathogen sensors, promptly engaged by several kinds of the so-called pathogen-associated molecular pattern (molecules) or PAMPs (if stresses are infectious in nature) or damage-associated molecular pattern (molecules) or DAMPs (if stresses are internal, such as Abs). This has been principally found relative to platelets (32, 33), and similar findings have been reported for erythrocytes (34). Furthermore, donor platelets express HLA class I Ags that differ in general from those of recipients. Donor cells are thus likely to be sensed as foreign by recipients’ circulating and vessel-lining leukocytes, which are prone to signaling this through a pro-inflammatory response, or by vessel endothelial cells. Experimental data suggest that endothelial cells can also signal the detection of foreign material by mounting a pro-inflammatory response (35–38). In general, though it is still difficult to link with certainty a host’s innate inflammatory response with unmanipulated donor cells, the danger theory of innate immunity would largely predict it in transfusion.

Recent data offer newer evidence supporting the hypothesis. First, a large Canadian clinical trial recently reported that age and sex of donors influenced the outcome of transfusion in recipients, more than any other factor (e.g., age of blood or pathology) (39, 40). Although there is now good evidence that there are differences between males and females in pathology and in particular in immune responses to infection or vaccines and inflammation processes, the gender issue has not been specifically addressed satisfactorily in transfusion medicine (41): this is perhaps a path for further investigation.

Our own investigations have shown that donors present great variation in the genes coding for CD40L; CD40L was investigated because platelets are the major purveyors of sCD40L in the body (42), and this BRM influences both innate and adaptive immunity (43). CD40L gene polymorphism was found to influence the presentation of secreted CD40L (44). It has been hypothesized that this genetic characteristic of donors may affect pro-inflammatory secretion of donated platelets in BCs (45, 46). This type of result is plausible as well for other BRMs.

### Blood Component Manufacturing and Storage Lesions with Pro-inflammatory Consequences in Recipients

A large body of reviews has documented this topic. We may consider two sets of data for illustrative purposes: one explores the secretory capacity of stored platelets over time or of platelets undergoing stress lesions upon collection, processing, and storage (47–50); the other explores the age of blood – and more precisely, the age of pRBCCs – at delivery. Both data sets incorporate diverse readouts: BRMs, oxidants, free iron, and extracellular vesicles (51–54). Despite such extracellular vesicles are reported to carry pro-inflammatory factors (55), some anti-inflammatory properties of extracellular vesicles have been reported (56), suggesting a fine-tune balance of inflammatory responses in relation of extracellular vesicles, likely depending on their sizes (57), origin, and abundance.

To summarize, it is generally reported that longer PC storage is accompanied by greater production of pro-inflammatory cytokines, which make up the majority of anti-inflammatory products (58–62) (Table 1). If leukocytes are still present in the PCs, leukocyte- and platelet-originating cytokines and other BRMs potentiate each other over time (63). Our own group has reported that there is a direct relationship between (i) the secretion of sCD40L [proved to exert a pathogenic effect in certain recipients, together with companion BRMs Ox40L and IL-27 (64, 65)] and the component shelf life; and (ii) between the net amount of sCD40L (alongside potentiating molecules IL-13 or MIP-1α) and the manifestation of an inflammatory AE in the recipient (62). Similar findings exist for mitochondrial DNA (66–68). In addition, it has also been proposed that the techniques used to obtain PCs influence pro-inflammatory reactions, as these techniques do not expose platelets to the same stress (62, 69). Together, these observations strongly suggest the platelet storage lesions have a role in PC induced inflammation and its balance in transfused patients.

**Table 1:** **Blood product storage and biological response modifier release**.

	Packed red blood cell concentrates	Platelet concentrates	Plasma for direct therapeutic use
Usual storage time	42 days	5 days	1 year
Main product transformation	Leucoreduction Irradiation Pediatric preparation Deplasmatization/washing Volume reduction Cryopreservation Reconstituted blood	Automated cell separation Centrifugation Leucoreduction Platelet additive solutions Occasionally pathogen Reduction or inactivation technology Irradiation Deplasmatisation/washing Cryopreservation Volume reduction	Leucoreduction Freezing/thawing Solvent-detergent Chemical and light pathogen inactivation Lyophilization
Lesion storage	Shape changes from a normal biconcave disk to echinocytes and spheroechinocytes ↑ Ammonium ↑ Free Hb in plasma ↑ K+ from ↓ ATP ↓ 2,3 DPG to <10% of original levels – replenished ↓ Labile proteins, e.g., complement, fibronectin, and coagulation factors ↓ to negligible ↓ Na+ ↓ pH ↓ NADH ↑ Bioactive substances (free Hb, hemin, microvesicles, iron, cytokines, lipids, and enzymes) ↓ S-nitrosohemoglobin (SNO-Hb) bioactivity	Shape changes from discoid to spheroid ↑ Activation (↑ release of granular contents) ↑ Proteolysis Altered platelet surface receptor expression ↑ Platelet aggregates Decreased mean platelet volume (MPV) ↑ Volume and density heterogeneity ↑ Procoagulant activity ↑ Platelet apoptosis ↓ pH, pO_2_, and glucose ↑ pCO_2_ ↑ Lactate production ↑ Glucose consumption ↓ Calcium ion flux ATP/ADP ratio change ↓ Mitochondrial oxidative respiration ↓ Fibrinogen binding	↑ Proteases ↑ Oxidation of Pro, Arg, Lys, Thr, Glu, or Asp side chains ↑ Cleavage of protein backbone ↑ Incorporation of lipid peroxidation products into Cys, His, or Lys residues ↑ Formation of advanced glycation end products ↑ Lipid peroxidation
Released/increased factor	MPs, IL-8, TNF-α, RANTES, NAP-2, Gro-α, MIP-1α, SDF-1, ENA-78, TGF-β, … Microvesicles	EGF, ENA-78, Gro-α, IL-1β, IL-6, IL-7, IL-8, IL-27, Lyso-PCs, sOX40L, PAI-1, PDGF-AA, PF4, RANTES, sCD40L, TGF-β, TNF-α, VEGF, β-TG, … Microvesicles Mitochondrial DNA	MPO, ECP, and histamine increase after thawing IL-1β, IL-4, and IL-10 increase with freeze/thaw cycles MMP-7 increases with the number of freeze/thaw cycles IL-4, IL-12, and TNF-α increase with the number of freeze/thaw cycles …

The situation for erythrocytes is even more complex. Substantial experimental evidence suggests that the age of erythrocytes, and the subsequent freeing of iron, is directly responsible for inflammation in experimental models, both *in vivo* and *ex vivo/in vitro* (70–74). Thus far, however, clinical trials have consistently failed to support this hypothesis (75). It should nevertheless be noted that this is extremely difficult to investigate and that further trials are necessary to resolve the matter (76).

### Consequences of Inflammation in Recipients: Manifestations of Adaptive Immunity

There are two main consequences of inflammation in blood component recipients. One – alloimmunization to foreign Ags – is rather clear. The other is transfusion-related immune modulation or TRIM. TRIM is a complex occurrence that involves a number of adaptive immune tools, of which suppressive CD8+ T cells, regulatory T (and probably B) cells, anti-idiotypic T cell clones, along with soluble HLA molecules and other supposed mediators (77–79). It must be made clear that, if the main visible consequence of immunization to foreign Ags is alloimmunization, there is a likely strong T cell immunity; however, it seems difficult to catch it up, and the majority of published works focus on Ab production. One may hypothesize that T cell immunity and TRIM rather explain cases where immunization is not productive in terms of Ab formation. Another consequence of TRIM is perhaps the likely depression of immune surveillance with the report of suspected increase of posttransfusion infections (that are quite well documented) and perhaps malignancies or organ dysfunctions (that are to be ascertained) (80–83).

Alloimmunization remains the most frequently reported AE of transfusion. It is often reported in pathologies where extended matching of red cells is difficult to achieve, for people needing repeated transfusions, such as sickle-cell disease or β-thalassemic patients. Indeed, as there are near 350 Ags on erythrocytes, a perfect match is unlikely. Recipient characteristics such as how well one presents HLA are also considered to have a major impact (84). In addition, residual leukocytes are highly potent immunizers (far more commonly in HLA groups than in HNA groups); platelets are also good immunizers in both HLA class I and HPA groups (85). However, it has been demonstrated that residual leukocytes can influence the global immunization score: as shown in experimental models, stringent but incomplete leucoreduction minimizes alloimmunization, while strict leucoreduction reinforces it, supposedly by erasing the TRIM effect (84).

Yet in spite of several attempts to decipher innate immune mechanisms acting as layers of inflammation that fuel Ag presentation, the details of alloimmunization largely remain a mystery (86).

Last, another consequence of inflammation is the reported enhanced erythrocyte phagocytosis by spleen cells. Inflammation created by excess iron and nitric oxide (NO) freeing – perhaps linked with aged erythrocytes – would aggravate anemia instead of correcting it by bringing Hb/O_2_ (87). While there is good experimental evidence in favor of this pathophysiology, clinical relevance is not yet ascertained.

## From Bench to Bedside: Paths to Improve Patients’ Safety

**Figure 1:**
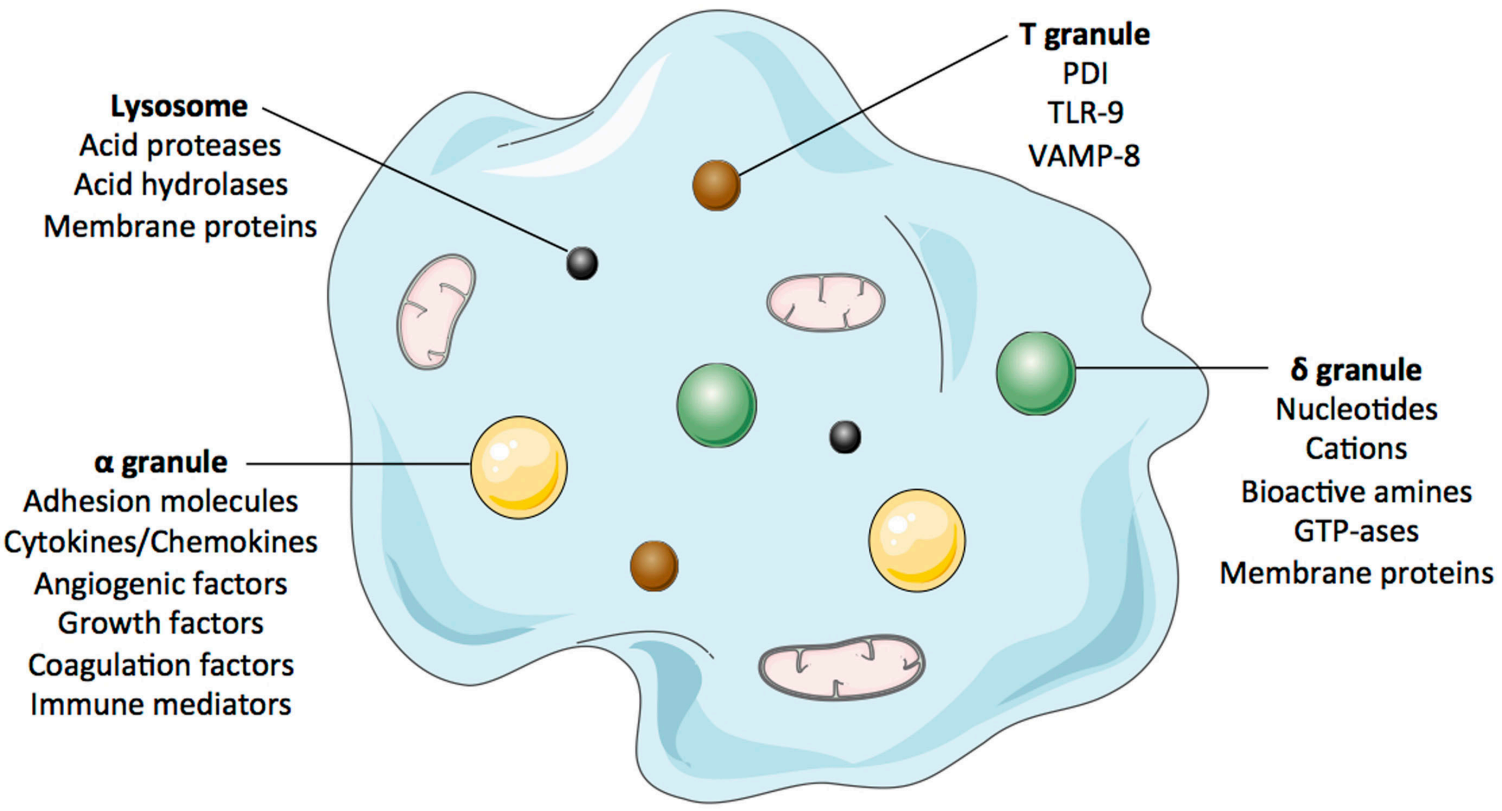
**The figure cartoons the platelet’ main granules and their secretory content**. Most products that can be released by platelets are listed in complementary Table 2.

Pathophysiological studies of platelets reveal that any exposure to stress may have consequences. Depending on the nature of this stress, platelets can mobilize predefined patterns of BRMs (88, 89). Indeed, contrary to what we might expect, given that they are anucleate, platelets do not indiscriminately release granule content through an all-or-nothing mechanism but rather exhibit stress-dictated processes (32, 90–93). This may explain why certain patients having received PCs manifest allergic reactions (where δ-granule BRMs predominate) or FNHTR/inflammation (where α-granule BRMs predominate) (Figure 1; Table 2). A readout in the PC leftover is given by the predominance of either IL-13 or MIP-1α in addition to sCD40L (62). Platelets are extremely reactive cells, and it is almost impossible to not pre-activate them while processing PCs for transfusion purposes. However, it has been made clear that the collection process, i.e., apheresis vs. recovered platelets from whole blood; platelets recovered from platelet-rich plasma vs. from buffy coats; PAS vs. plasma; pathogen reduction/inactivation vs. no additional safety measure; and in the case of apheresis, type of cell separator – and the length of storage are important parameters to control activation (59, 62, 65, 94–97). Despite conflicting data (98), it cannot be ruled out that ABO compatibility vs. identity also affects the outcome of platelet transfusion (99, 100), but probably not by triggering pre-activation. Furthermore, PCs are almost always HLA incompatible, at least for the majority of expressed class I Ags. This has not appeared to be deleterious in terms of clinical outcome – in the PLADO trial (98), for example, but consistent recommendations suggest that refractoriness to platelet transfusion is better addressed with HLA-compatible PCs (97). The HPA case is barely addressed, unless a specific and pathogenic Ab is identified, or in the case of fetal/neonatal maternal incompatibility (101). Serious allergies, allergic reactions, or severe FNHTRs can be addressed – when further PC transfusions are needed – by washing the components. This process is nevertheless tedious as it may itself pre-activate the cells. Alternatively, some teams may absorb pathogenic BRMs on columns when available (102, 103). At present, there are parameters that cannot be controlled (recipients’ genetic characteristics and – to a large extent – donors’ characteristics) and those that can be partly controlled, i.e., manufacturing, ABO matching, and aging of the PCs (Table 3). Most efforts appear to focus on the last three issues to limit inflammation in recipients. Further investigations are needed to evaluate the actual impact of safety measures in PC recipients and determine whether efforts can be made to propose matching procedures that can calculate the most important factors to limit inflammatory responses in patients.

**Table 2:** **Platelet granule main contents**.

α granules	δ granules	T granules	Lysosomes
Adhesion molecules αIIbβ3, αVβ3, CD9, fibronectin, GPIbα, multimerin, osteonectin, PECAM, P-selectin, vitronectin, vWF Cytokines/chemokines β-thromboglobulin, CCL4, CCL17, ENA-78, Gro-α, IL-1, IL-7, IL-8, MCP-1, MCP-3, MIP-1α, NAP-2, PF4, RANTES, sCD40L, SDF-1 Angiogenesis/growth factors ADAM10, ADAMTS13, angiostatin, angiopoietin-1, BDNF, bFGF, BMP-2, BMP-4, BMP-6, CTAP-III, CTGF, EGF, endostatin, HGF, HGR, IGF-1, kininogen, MMP-1, MMP-2, MMP-9, PDGF, TGF-β, thrombospondin, TIMP-1, TIMP-4, VEGF Coagulation factors α_2_-antiplasmin, α_2_-antitrypsin, α2-macroglobulin, antithrombin, factor V–VIII–XI–XIII, fibrinogen, PAI-1, plasmin, plasminogen, protease nexin-2, protein S, prothrombin, TFPI Immune mediators β1H globulin, C1 inhibitor, complement factors, factor D, IgA, IgG, IgM, platelet factor H, thymosin-β4 Others Albumine, PDCI	Nucleotides ADP, ATP Cations Calcium, magnesium Bioactive amines Serotonin, histamine GTP-ases rab27a, rab27b Membrane proteins αIIbβ3, CD63, GPIb, LAMP-1, LAMP-2, P-selectin Others Polyphosphate, pyrophosphate	PDI TLR-9 VAMP-8	Acid proteases Acid phosphatase, arylsulphatase Carboxypeptidase A–B, cathepsin D–E, collagenase, elastase, proline carboxypeptidase Acid hydrolases α-arabinofuranosidase, α-fucosidase, β-fucosidase, α-galactosidase, β-galactosidase, β-glucoronidase, α-mannosidase, α-glucosidase, β-glucosidase, β-*N*-acetyl-hexosaminidase Membrane proteins CD63, LAMP-1, LAMP-2

*ADP, adenosine diphosphate; ATP, adenosine triphosphate; BDNF, brain-derived neurotrophic factor; bFGF, basic fibroblast growth factor; BMP, bone morphogenetic protein; C, complement; CTAP-III, connective tissue-activating peptide III; CTGF, connective tissue growth factor; EGF, epidermal growth factor; HGF, hepatocyte growth factor; HGR, histidine-rich glycoprotein; Ig, immunoglobulin; IGF, insulin-like growth factor; IL, interleukin; LAMP, lysosomal-associated membrane protein; MCP, monocyte chemotactic protein; MIP, macrophage inflammatory protein; MMP, matrix metalloproteinase; NAP, neutrophil-activating protein; PAI, plasminogen activator inhibitor; PDCI, platelet-derived collagenase inhibitor; PDGF, platelet-derived growth factor; PDI, protein disulfide isomerase; PECAM, platelet endothelial cell adhesion molecule; PF, platelet factor; RANTES, regulated on activation normal T cell expressed and secreted; SDF, stromal cell-derived factor; TFPI, tissue factor pathway inhibitor; TGF, transforming growth factor; TIMP, tissue inhibitor of metalloproteinases; TLR, toll-like receptor; VEGF, vascular endothelial growth factor; VAMP, vesicle-associated membrane protein; vWF, von Willebrand factor*.

**Table 3:** **Examples of preventable and not yet preventable causes of inflammation in transfusion medicine**.

	Parameters that can be addressed	Parameters that cannot yet be addressed
Donor-related parameters	– So-called irregular antibodies to red blood cells or HLA – Autoantibodies – Potentially: allergens and IgE antibodies to allergens – Infectious pathogens and infectious pathogen-derived material (toxins, residues, superantigens) – …	Genetic parameters predisposing to inflammation
Processed component-related parameters	– Typically: leukocytes – Microvesicles/microparticles – All types of storage lesions – Age of blood^a^ – …	
Recipient-related parameters	– Certain therapies (drugs)	– Genetic parameters that predispose to inflammation – Clinical state (causal disease or treatment being the cause of the transfusion need) – Most therapies, otherwise needed – Preexisting alloimmune Abs, autoimmune Abs – …
Standard of operation parameters (SOP)	– Main blood group matching – Blood component freshness^a^	– Fine-tuned blood group matching

*^a^Age of blood appears to fall into either category as it affects the release of biological response modifiers (storage lesions) and likely sustains TRIM, and it affects the release of, e.g., oxygen (SOP) and the recirculation or cells (and propensity to apoptosis or to be prone to phagocytosis)*.

For pRBCCs, the appropriate strategy may be very simple or very complex. In theory, it should be simple if one considers that the enemy is alloimmunization, which concerns inflammation. To limit the risk of immunization, an improvement of blood group Ag matching would be ideal; however, considering the volume of blood components to be issued to millions of recipients, this is simply not achievable on a routine basis. Efforts are being made to facilitate matching for at-risk recipients, such as those routinely receiving transfusions, though success varies according to specific needs of ethnic groups transfused outside their native region, where erythrocyte Ag group distribution differs from their own. Further, if inflammation fuels alloimmunization, some genetic control of responders vs. non-responders – or more precisely, good vs. bad HLA presenters – seems to prevail: this has been observed for certain blood group Ags and is very likely true for all others (77, 104). The situation is more complex than for platelets because the triggers of inflammation are less clearly identified. The age of blood is a likely but unproven factor, and the effects of storage lesions and erythrocyte Ag alloimmunization (105). Various teams have provided indirect evidence after examining whether RBC collection can stress RBCs and subsequently stress endothelial cells exposed to such RBCs (at least in *ex vivo/in vitro* models) (106, 107). Here again, until more direct evidence becomes available, one may heed protocols that minimize stress to donors’ RBCs and subsequently to recipients’ vascular endothelium. Accordingly, some authors recommend not overexposing cellular blood components to radiation unless absolutely required as this may increase storage lesions (54, 108, 109). Closer examination is needed to determine the extent to which irradiation of BCs favors alloimmunization. Similar caution has been suggested for pathogen inactivation/reduction technologies, but there are conflicting claims in favor of reduction or alloimmunization based on impairment of indirect Ag presentation (84). This too calls for further investigation.

## Conclusion

Transfusion is an old therapy, though it is not obsolete. In fact, it is quite modern if seen as cell therapy or biotherapy (110). It is very commonly used and is nowadays associated with few nosocomial AEs. Moreover, not all AEs are truly nosocomial as some are in fact linked to characteristics of recipients that can neither be dampened nor counteracted by matching blood components. When transfusion is associated with AEs, most can be related to an inflammatory state, which is either obvious (allergy, FNHTR, hypotension) or ascribed to such a state by current knowledge (alloimmunization). Indeed, transfusion-transmitted infection has become a rarity, and novel means are regularly applied to further minimize their occurrence. Means of decreasing the occurrence of transfusion-associated inflammation have received less attention and care, though they should be our new focus, to help patients, secure resources, and limit indirect costs. Platelet pathophysiology owes a lot to transfusion medicine: many of the major discoveries in this field were made by researchers who, questioning the role of PC transfusion in AEs (and occasionally SAEs), attempted to solve questions about platelet activation and secretion. This review has not considered the other side of the coin with respect to platelets and their role in the inflammation process. Namely, in addition to being the source of many pro-inflammatory BRMs, platelets also produce healing factors (the terminus of physiological inflammation) that may also be used as therapeutic tools (111).

## Author Contributions

OG drafted the manuscript; all other contributors contributed illustrations, discussion, and critical review, along with the production of original data supporting the synthesis as a review article.

## Conflict of Interest Statement

The authors declare no competing financial interests and no conflicts of interest regarding this study. The reviewer PS declared a shared affiliation, though no other collaboration, with one of the authors FC to the handling Editor, who ensured that the process nevertheless met the standards of a fair and objective review.
